# The expression of the β-defensins hBD-2 and hBD-3 is differentially regulated by NF-κB and MAPK/AP-1 pathways in an *in vitro *model of *Candida *esophagitis

**DOI:** 10.1186/1471-2172-10-36

**Published:** 2009-06-12

**Authors:** Nadine Steubesand, Karlheinz Kiehne, Gabriele Brunke, Rene Pahl, Karina Reiss, Karl-Heinz Herzig, Sabine Schubert, Stefan Schreiber, Ulrich R Fölsch, Philip Rosenstiel, Alexander Arlt

**Affiliations:** 1 Department of Internal Medicine, University Hospital Schleswig-Holstein, Campus Kiel, Kiel, Germany; 2 Institute of Clinical Molecular Biology, University Hospital Schleswig-Holstein, Campus Kiel, Kiel, Germany; 3 Department of Department of Dermatology and Allergology; University Hospital Schleswig-Holstein, Campus Kiel, Kiel, Germany; 4 Institute of Biomedicine, Div of Physiology, and Biocenter of Oulu, University of Oulu, and Department of Internal Medicine, University of Kuopio, Finland; 5 Institute for Infection Medicine; University Hospital Schleswig-Holstein, Campus Kiel, Kiel, Germany

## Abstract

**Background:**

*Candida albicans *resides on epithelial surfaces as part of the physiological microflora. However, under certain conditions it may cause life-threatening infections like *Candida *sepsis. Human β-defensins (hBDs) are critical components of host defense at mucosal surfaces and we have recently shown that hBD-2 and hBD-3 are upregulated in *Candida *esophagitis. We therefore studied the role of *Candida*te signalling pathways in order to understand the mechanisms involved in regulation of hBD-expression by *C. albicans*. We used the esophageal cell line OE21 and analysed the role of paracrine signals from polymorphonuclear leukocytes (PMN) in an *in vitro *model of esophageal candidiasis.

**Results:**

Supernatants of *C. albicans *or indirect coculture with *C. albicans *induces upregulation of hBD-2 and hBD-3 expression. PMNs strongly amplifies *C. albicans-*mediated induction of hBDs. By EMSA we demonstrate that *C. albicans *activates NF-κB and AP-1 in OE21 cells. Inhibition of these pathways revealed that hBD-2 expression is synergistically regulated by both NF-κB and AP-1. In contrast hBD-3 expression is independent of NF-κB and relies solely on an EGFR/MAPK/AP-1-dependent pathway.

**Conclusion:**

Our analysis of signal transduction events demonstrate a functional interaction of epithelial cells with PMNs in response to *Candida *infection involving divergent signalling events that differentially govern hBD-2 and hBD-3 expression.

## Background

*Candida albicans *colonize distinct microanatomical regions such as the oro-gastointestinal tract as a commensal, but also accounts for more than 50% of all fungal systemic infections [1]. *Candida *esophagitis represents a severe threat to an immunocompromised body and, especially in neutropenic patients, often is the first manifestation before *Candida *sepsis develops [2,3]. Host defense mechanisms preventing mucosal (e.g. esophageal) candidiasis are poorly understood, but include both innate and adaptive responses [4,5].

The gastrointestinal epithelial layer represents a barrier that is usually adequate to restrain commensal microbes, but is often insufficient to protect against microbial pathogens. Once this physical barrier is penetrated, recognition of invading microbiota is the first step in the initiation of a fast immune response and involves the activation of pattern recognition receptors by microbial pathogens and their products [6-8]. *C. albicans *have been shown to activate a subset of pattern recognition receptors, the Toll-like receptors (TLRs). This family of transmembrane receptors recognizes a broad variety of signature motifs on microbes and transduces signals leading to the activation of transcription factors [9], production of cytokines and antimicrobial peptides [10,11]. However there is some controversy and inconclusive data which TLR subtypes are activated by *C. albicans*. Some reports indicated a critical role for TLR2 and TLR4 in activating the host defense response alone or in combination with the ss-glucan receptor Dectin-1,[5,12,13] whereas other studies showed that TLR-1 and TLR-6 are responsible for the recognition of *C. albicans *[14,15].

In addition the downstream effectors in the immune response against *C. albicans *are largely unknown. There is growing evidence that human β-defensins (hBDs) are critical components of both the innate and adaptive immune responses to *Candida *infections with distinct antifungal efficacies and mechanisms for hBD-2 and hBD-3 [16-19]. Expression of hBDs is regulated by a plethora of proinflammatory cytokines like IL-1β, TNF-α and EGF-receptor ligands activating downstream effectors like the transcription factors NF-κB or AP-1 [20-22]. hBDs are secreted by epithelial cells and protect the gastrointestinal mucosa by antimicriobial activity as well as by chemotactic properties recruiting polymorphonuclear leukocytes (PMN) to the site of infection [21,23]. Transepithelial migration of PMNs is observed during oral *Candida *infection and is believed to play a crucial role in the clearance of infection and in epithelial homeostasis [5,24]. This protective phenotype is associated with production of epithelial proinflammatory cytokines, including IL-8, IL-1β and TNF-α. However, the mechanisms by which PMNs and epithelial cells interact to protect mucosal surfaces from *C. albicans *invasion are mostly unknown.

We have recently shown that hBD-2 and hBD-3 are highly expressed in *Candida *esophagitis and that the α-defensins Human Neutrophil Peptides 1–3 were also upregulated [25] indicating involvement of neutrophils in the immune response to the *C. albicans *infection [26]. This is in line with the observation that IL-8 is strongly expressed in the mucosa of patients suffering by *Candida *infection [25], since this cytokine is involved in recruitment of polymorphonuclear leukocytes (PMNs) to sites of microbial infection [27].

In the present study we explored the mechanisms involved in the induction of hBD expression in an *in vitro *model of *Candida *esophagitis. Using this co-culture model we analyzed the contribution of PMNs in the regulation of epithelial hBD expression. We found that hBD-2 and hBD-3 are strongly upregulated through high concentrations of *C. albicans *alone. If lower concentrations of *C. albicans *were used, only a moderate induction of these hBDs were observed that was strongly enhanced by coculture of the epithelial cell line with PMNs, emphasizing the important role of mesenchymal-epithelial interactions in early host defense against fungal infection. Furthermore we were able to show that the NF-κB and MAPK signalling pathways contribute to the regulation of hBD-2 and that hBD-3 is a downstream target of a distinct EGFR/MAPK/AP-1 pathway in *Candida *esophagitis.

## Results

### Supernatants of *C. albicans *induce hBD expression in OE21 cells

**Figure 1:**
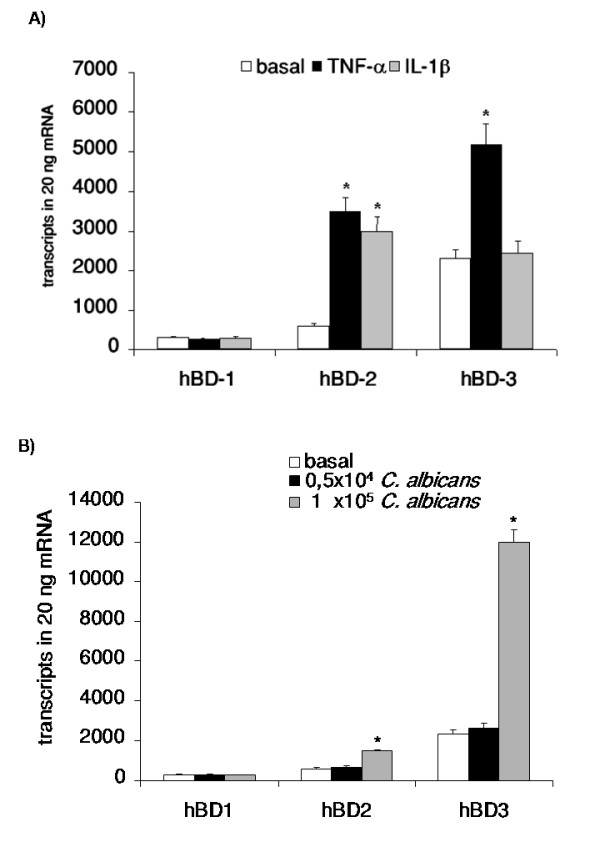
**Supernatants of *C. albicans *induce hBD expression in OE21 cells**. OE21 cells were stimulated with A) TNF-α (20 ng/ml), IL-1β (20 ng/ml) or B) supernatants of 0.5 × 10^4 ^C. albican/ml or 1 × 10^5 ^C. albican/ml for 24 h. hBD-1, hBD-2 and hBD-3 gene expression was assessed by real time RT-PCR. Means ± s.d. of three independent experiments performed in triplicate are shown, * indicates p-value <0,05.

To establish an in vitro model for *Candida *esophagitis we first treated several oesophageal cell lines (i.e. OE21, KYSE70, KYSE7150, KYSE7180, KYSE410 and Colo680N) with established inducers of hBD-expression. Only for the OE21 cell line a reliable induction of the mRNA of hBD-2 and hBD-3 could be detected (Figure 1A). We next challengend OE21 cells with supernatants of *C. albicans *cell culture growing under various concentrations of *C. albicans*. Supernatants of *C. albicans *induce upregulation of hBDs in a concentration-dependent manner. For the supernatants of the density of 0.5 × 10^4 ^*C. albicans *cells/ml no significant induction of hBD-2 and hBD-3 was detected (Figure 1B). In contrast the supernatants of 1 × 10^5 ^*C. albicans *cells/ml resulted in a significant upregulation of hBD-3 and to a smaller extent of hBD-2 (Figure 1B). hBD1 expression was not altered by one of the inducers or the supernatants (Figure 1).

### PMNs significantly enhances hBD-2 and hBD-3 expression during *C. albicans *infection

**Figure 2:**
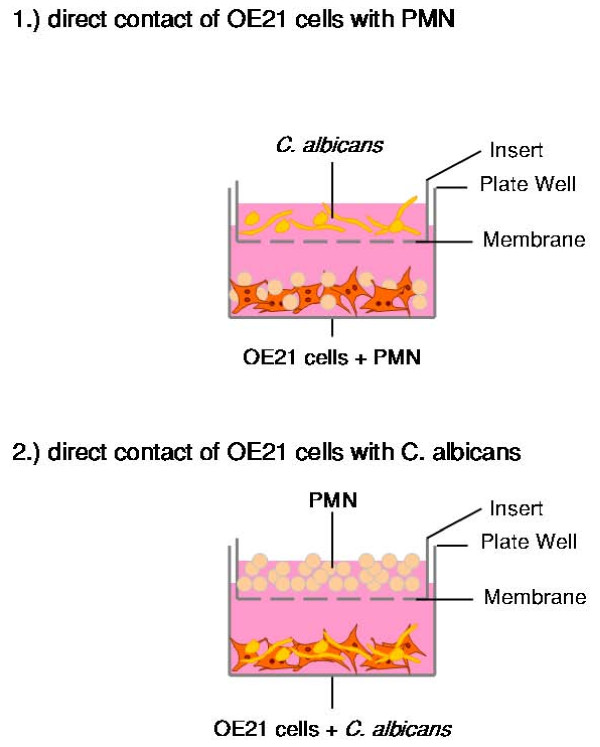
**PMNs enhances hBD-2 and hBD-3 expression during *C. albicans *infection**. OE21 cells were stimulated with 0.5 × 10^4 ^C. albican/ml or 0.5 × 10^6 ^PMNs/ml alone or indicated combinations in a transwell setting for 24 h. Shematic presentation of experimental setting.

**Figure 3:**
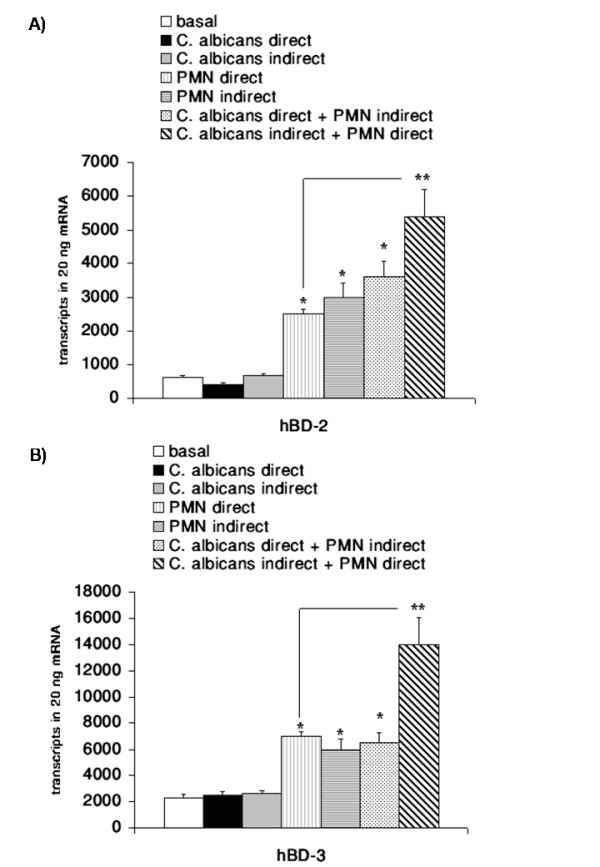
**PMNs enhances hBD-2 and hBD-3 expression during *C. albicans *infection**. OE21 cells were stimulated with 0.5 × 10^4 ^C. albican/ml or 0.5 × 10^6 ^PMNs/ml alone or indicated combinations in a transwell setting for 24 h. A) hBD-2 and B) hBD-3 gene expression was assessed by real time RT-PCR. Means ± s.d. of three independent experiments performed in triplicate are shown, * indicates p-value <0,05, ** indicates synergistic effects with a p-value <0,05.

To elucidate a potential functional interaction of PMNs and epithelial cells in the immune response to *C. albicans *infection we established a transwell coculture model. In this two-compartment model we tested the effect of a coculture of OE21 cells, PMNs from healthy donors and *C. albicans *in different settings: 1) direct contact of OE21 cells with PMNs and 2) direct contact of OE21 cells with *C. albicans*. The corresponding third component of the system (i.e. *C. albicans *or PMNs) was cocultured in the transwell system allowing for the interaction of auto- and paracrine factors (Figure 2). Since higher cell numbers than 0.5 × 10^4 ^*C. albicans *cells/ml or 0.5 × 10^6 ^PMN/ml repressed growth of OE21 cells in the direct setting (data not shown), all experiments were conducted with these concentrations of *C. albicans *and/or PMNs. The direct or indirect coculture of OE21 with *C. albicans *had only small effects on the expression of hBD-2 (Figure 3A) and hBD-3 (Figure 3B) comparable with effects observed with supernatants of the low concentration of *C. albicans *(Figure 1B). The direct interaction of OE21 cells with PMNs led to an increase of the expression of both hBDs (Figure 3A and Figure 3B). The interaction of OE21 cells with PMNs lead to a significant upregulation of *C. albicans *induced hBD-2 and hBD-3 expression compared to the induction with *C. albicans *or PMN alone (Figure 3A and Figure 3B). Substitution of the live *C. albicans *by supernatants of the density of 0.5 × 10^4 ^*C. albicans *cells/ml also lead to the synergistic effects. To keep the manuscript mor concise we only show the data for the live *C. albicans*.

### *C. albicans *activate NF-κB in OE21 cells

**Figure 4:**
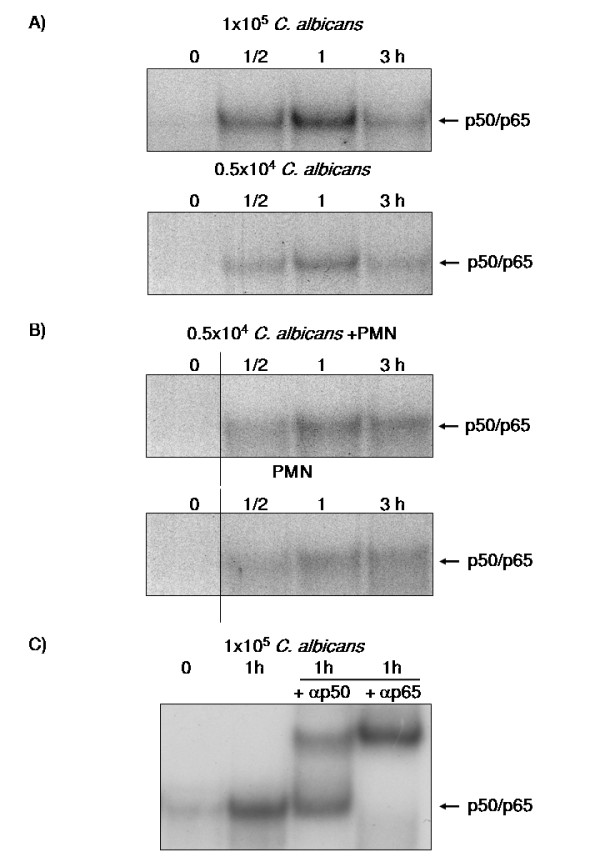
***C. albicans *activate NF-κB in OE21 cells**. OE21 cells were stimulated with A) 0.5 × 10^4 ^C. albican/ml, 1 × 10^5 ^C. albican/ml, or B) 0.5 × 10^6 ^PMNs/ml alone or with direct contact with 0.5 × 10^6 ^PMNs/ml and coincubation with 0.5 × 10^4 ^C. albican/ml in a transwell setting for indicated time periods. Nuclear extracts of these cells were submitted to EMSA using a radiolabelled probe for NF-κB. The arrow indicates the p50/p65 heterodimer. C) Supershift experiments with antibodies directed against the p50 or p65 subunit of NF-κB on nuclear extracts of OE21 cells treated for 1 h with 1 × 10^5 ^C. albican/ml.

To further analyse the mechanisms involved in the regulation of hBD expression during *Candida *esophagitis we determined whether the observed induction of hBD-2 and hBD-3 expression by *C. albicans *was associated with the activation of NF-κB. This transcription factor has been shown to be a central regulator of hBD-2 expression [22,28,29] but there is some controversy if NF-κB is also involved in the regulation of hBD-3 expression [20,30,31]. By EMSA we could show that NF-κB is activated (Figure 4) in association with *C. albicans *mediated induction of β-defensin expression. In parallel to the observed dependency of defensin expression on the cell number of *C. albicans *a concentration dependent activation of NF-κB was observed (Figure 4A). The coculture of OE21 cells with PMNs or *C. albicans *also led to an increased DNA-binding of NF-κB that was further enhanced in the transwell setting (direct PMN and *C. albicans *indirect; Figure 4B). Supershift assays revealed that the NF-κB complex consisted of the p50 and p65 subunits (Figure 4C).

### NF-κB is involved in the induction of hBD-2 but not hBD-3 expression

**Figure 5:**
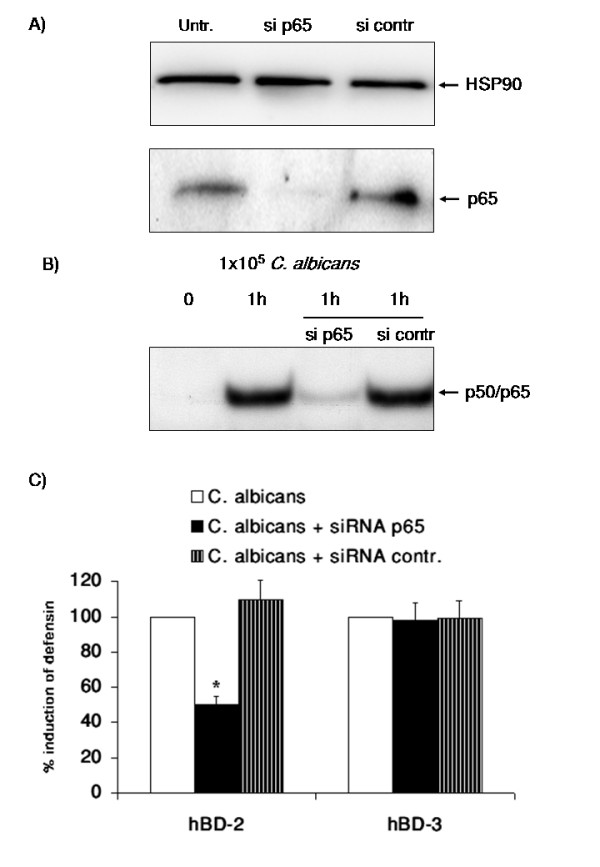
**NF-κB is involved in the induction of hBD-2 but not hBD-3 expression**. OE21 cells were transfected with a control siRNA or a siRNA directed against RelA/p65. A) Total protein extracts were submitted to Western Blotting for the analysis of RelA/p65 using HSP90 for normalization and B) EMSA on nuclear extracts of siRNA transfected OE21 cells treated 9 with 1 × 10^5 ^C. albican/ml for 1 h. Representative results from three independent experiments are shown. C) SiRNA transfected OE21 cells were incubated with 1 × 10^5 ^C. albican/ml for 24 h and hBD gene expression was assessed by real time RT-PCR. Means ± s.d. of three independent experiments performed in triplicate are shown, * indicates p-value <0,05.

To evaluate the putative contribution of NF-κB in *C. albicans *induced hBD expression we transiently transfected cells with siRNA directed against RelA/p65 as the transcriptional active subunit of the p65/p50 NF-κB complex [32]. Transfection of OE21 cells led to a significant reduction of the expression of RelA/p65 (Figure 5A). Furthermore the induction of NF-κB by *C. albicans *was inhibited by this strategy as shown by EMSA (Figure 5B). This reduction of NF-κB activation strongly reduced the *C. albicans *mediated induction of hBD-2 expression but had no effect on the hBD-3 expression (Figure 5C). In the coculture settings the reduction of RelA/p65 expression also attenuated hBD-2 induction (data not shown). These results support a critical role of NF-κB in regulating hBD-2 but not hBD-3 expression.

### Role of AP-1 and the MAPK Pathway in *C. albicans *mediated β-defensin induction

**Figure 6:**
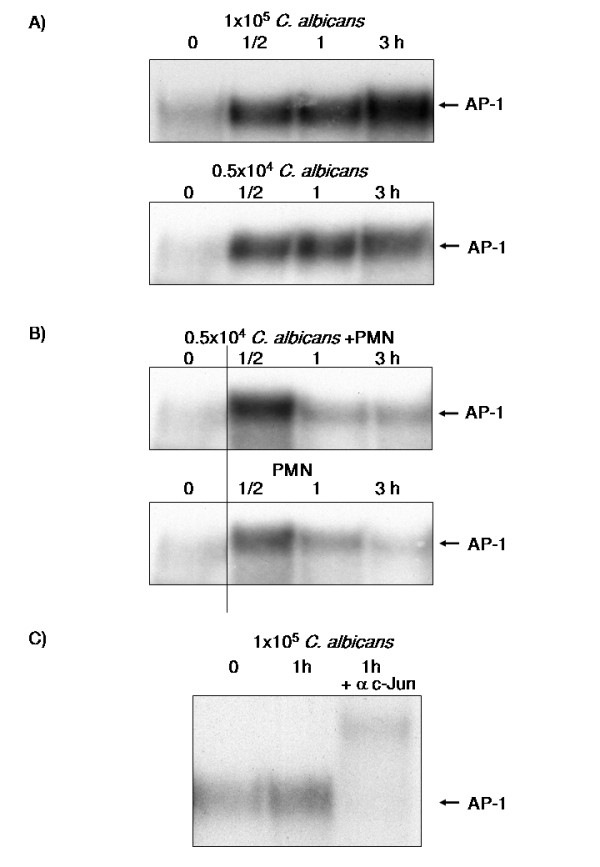
**Role of AP-1 and the MAPK Pathway in *C. albicans *mediated hBD induction**. OE21 cells were stimulated with A) 0.5 × 10^4 ^C. albican/ml, 1 × 10^5 ^C. albican/ml, or B) 0.5 × 10^6^PMNs/ml alone or with direct contact with 0.5 × 10^6 ^PMNs/ml and coincubation with 0.5 × 10^4 ^C. albican/ml in a transwell setting for indicated time periods. Nuclear extracts of these cells were submitted to EMSA using a radiolabelled probe for AP-1. The arrow indicates the AP-1 complex. C) Supershift experiments with an antibody directed against the c-Jun subunit of AP-1 on nuclear extracts of OE21 cells treated for 1 h with 1 × 10^5 ^C. albican/ml.

Our results indicated a critical role for NF-κB in *C. albicans *mediated induction of hBD-2 but not for the regulation of hBD-3 expression. Since the hBD-3 promotor contains a functional binding site for the AP-1 transcription factor [33] we conducted EMSA with a probe containing an AP-1 consensus sequence. *C. albicans *alone (Figure 6A) or in the coculture setting (Figure 6B) activated AP-1 in OE21 cells and supershift experiments identified c-jun as one of the central subunits involved (Figure 6C). Activation of the c-jun/AP-1 complex by the MAPK pathway is well established [34,35] and a recent report indicated a role of this pathway in the response of macrophages to the infection with *C. albicans *[9]. To analyse the contribution of the MAPK/AP-1 pathway in the induction of β-defensin induction by *C. albicans *pharmacological inhibitors selective for individual pathway were utilized.

**Figure 7:**
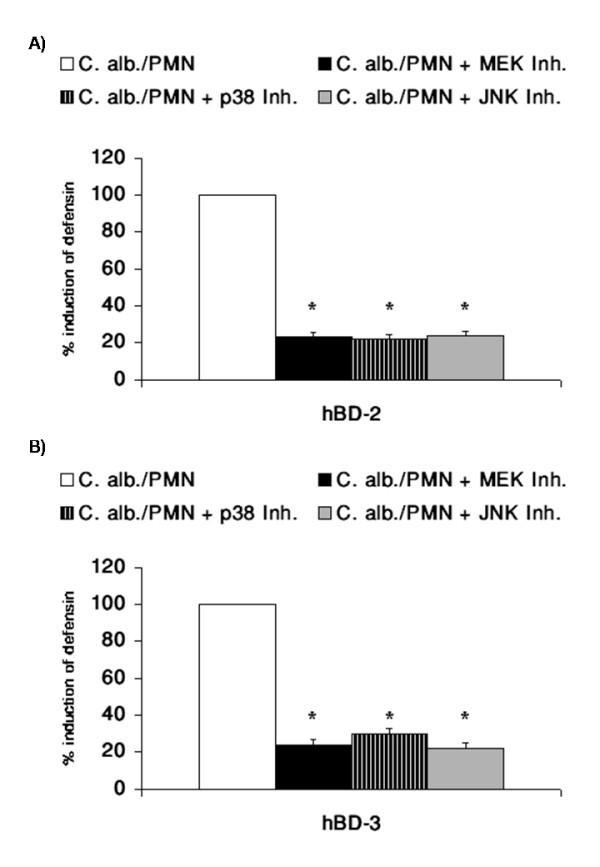
**Role of AP-1 and the MAPK Pathway in *C. albicans *mediated hBD induction**. OE21 cells were preincubated with inhibitors for MEK (PD98059, 10 μM), p38 (SB203580 10 μM) and JNK (SP600125 20 μM) kinase pathways for 1 h and then incubated with direct contact with 0.5 × 10^6 ^PMNs/ml and coincubation with 0.5 × 10^4 ^C. albican/ml in a transwell setting for additional 24 h. A) hBD-2 and B) hBD-3 gene expression was assessed by real time RT-PCR. Means ± s.d. of three independent experiments performed in triplicate are shown, * indicates p-value <0,05.

As shown in Figure 7A and 7B inhibition of each of the major MAPK pathways resulted in a reduction of hBD-2 and hBD-3 induction by *C. albicans*.

### Activation of the EGFR is an upstream event in regulation of hBD-3 expression

**Figure 8:**
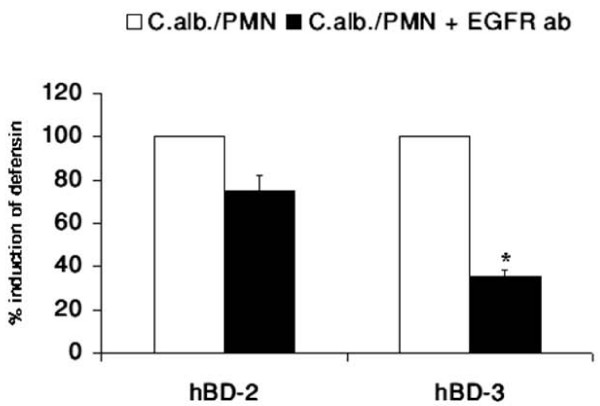
**Activation of the EGFR is an upstream event in regulation of hBD-3 expression**. OE21 cells were preincubated with a blocking EGFR antibody (10 μg/ml) for 1 h and then incubated with direct contact with 0.5 × 10^6^ PMNs/ml and coincubation with 0.5 × 10^4 ^C. albican/ml in a transwell setting for additional 24 h. hBD gene expression was assessed by real time RT-PCR. Means ± s.d. of three independent experiments performed in triplicate are shown, * indicates p-value <0,05.

Upstream of the MAPK pathway several growth factor receptors have been shown to be involved in the immune response to microbiota [20,36,37]. To investigate the upstream events leading to the activation of the MAPK pathway in infection with *C. albicans *we used blocking antibodies against several growth factor receptors. The results revealed a clear involvement of the EGF-receptor in the regulation of hBD-3 expression by *C. albicans*. Whereas induction of hBD-2 expression was slightly inhibited by a neutralizing antibody targeting EGFR transactivation, a significant reduction of *C. albicans*-induced hBD-3 expression observed (Figure 8).

### TGF-α is involved in EGFR mediated hBD-3 induction

**Figure 9:**
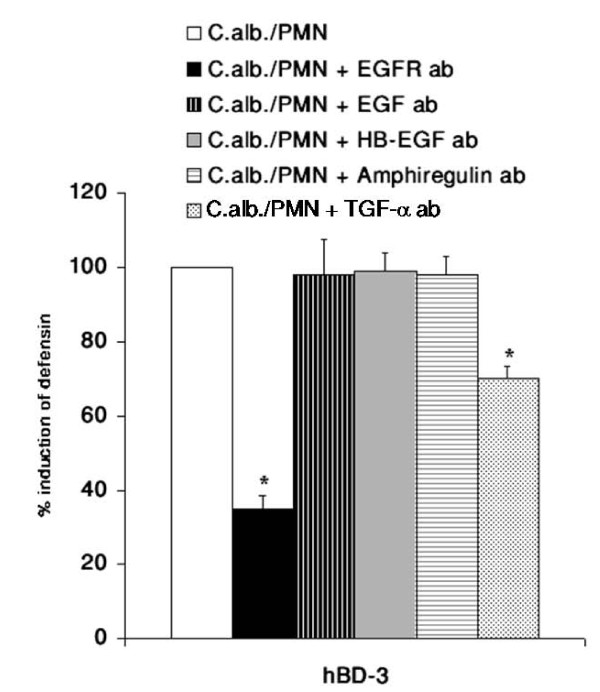
**TGF-α is involved in EGFR mediated hBD-3 induction**. OE21 cells were preincubated with the blocking EGFR antibody (10 μg/ml) or neutralizing antibody against EGF (0,5 μg/ml), HB-EGF (10 μg/ml), amphiregulin (10 μg/ml) or TGF-α (1 μg/ml) for 1 h and then incubated with direct contact with 0.5 × 10^6 ^PMNs/ml and coincubation with 0.5 × 10^4 ^C. albican/ml in a transwell setting for additional 24 h. hBD gene expression was assessed by real time RT-PCR. Means ± s.d. of three independent experiments performed in triplicate are shown, * indicates p-value <0,05.

Finally we searched for the ligand activating the EGFR. By using blocking antibodies for the established EGFR-ligands EGF, HB-EGF, amphiregulin and TGF-α we were able to show that TGF-α is involved in the EGFR mediated upregulation of hBD-3 expression (Figure 9).

## Discussion

*Candida *esophagitis represents a severe threat to an immunocompromised body and the course of the infection is determined by both pathogen- and host-dependent factors [15,24,38,39]. It is well established that epithelial cells of the esophagus are the central target of an oro-esophageal invasive *Candida *infection but there are only very limited data on the host response preventing a *Candida *esophagitis. In the present study we identified the NF-κB and MAPK/AP-1 pathways as central regulators of epithelial hBD-2 and hBD-3 expression during *C. albicans *infection. Furthermore we were able to show a crucial role of the interaction of PMNs with the epithelial cell compartment for the induction of hBD-2 and hBD-3 expression during *Candida *infection of esophageal epithelial cells. Finally hBD-3 expression is dependent on transactivation of EGFR by TGF-α. This is the first report delineating molecular mechanisms leading to upregulation of hBD-2 and hBD-3 in esophageal *Candida *infection suggesting differential regulation of these two central epithelial antibiotic peptides in response to a *C. albicans *infection. We were able to show a concentration dependency of hBD-2 and hBD-3 induction upon stimulation with *C. albicans *supernatants (Figure 1B). This dose dependent effect could be at least in part be mediated through stronger induction of NF-κB (Figure 4) and AP-1 (Figure 6) through higher concentrations of *C. albicans*. A comparable concentration-dependent effect of supernatants of environmental airborne fungi on cytokine release of eosinophils [40] and of *C. albicans *on the activation of NF-κB [9] was recently reported.

There is some controversy about the relevance of the NF-κB and AP-1 binding sites in the promotor of hBD-2 for full induction of hBD-2 expression after treatment with IL-1β or infection with microbiota. Wehkamp and collegues showed that the parallel activation of NF-κB and AP-1 is needed for full transcriptional activation of the hBD-2-promotor after IL-1β stimulation, treatment of keratinocytes with supernatants of *Pseudomonas aeruginosa *[22], or *E. coli *mediated hBD-2 induction [41]. On the other hand another study demonstrated induction of hBD-2 expression through *Fusobacterium nucleatum *in human gingival epithelial cells independent of NF-κB activation [42] and there is also evidence for induction of hBD-2 through Salmonella enteritidis [43] or H. pylori [44] in the absence of functional AP-1. In the present work we were able to show that both NF-κB and AP-1 activation are required for full upregulation of the hBD-2 mRNA after treatment with either supernatants of *C. albicans *or the coculture of *C. albicans *with PMNs. Inhibiton of the NF-κB or of the MAPK/AP-1 pathway significantly reduced the induction of hBD-2 expression confirming the central role of both transcription factors. These observations are in line with recent results for H. pylori infection [20] and the effects of lactobacilli and the VSL#3 bacterial mixture on enterocytes [45]. The role of NF-κB is less clear in the regulation of hBD-3 expression. In contrast to a recently reported role of NF-κB in the regulation of the hBD-3 gene in keratinocytes [31] the majority of data failed to show a functional relevance for NF-κB in controlling hBD-3 expression [33,46]. By using siRNA targeting the RelA/p65 subunit of NF-κB we could demonstrate that *C. albicans *mediates hBD-3 upregulation through a MAPK/AP-1 pathway independently of the observed NF-κB acitvation. To investigate the proposed role of PMNs in the immune response the *C. albicans *infection [25,26] we established an in vitro model (Figure 2). PMNs alone induced NF-κB and AP-1 leading to hBD-2 and hBD-3 expression. Coincubation of PMNs with *C. albicans *lead to a significant upregulation of hBD-2 and hBD-3 expression compared to effects of PMNs or *C. albicans *alone. Inhibition of the MAPK/AP-1 pathway reduced the expression of both hBDs under this condition. The fact that PMNs alone induced hBD expression indicated that PMNs could contribute to the induction of antimicrobial peptides in epithelia during inflammation. There is some evidence that a TLR-4 mediated interplay between PMNs and epithelial cells is important for the protective response against *Candida *infections [5] but the exact mechanisms remained elusive. One the one hand PMNs induced expression and release of IL-6 and IL-8 in O21 cells (Additional file 1) which might in turn activate NF-κB and AP-1 leading to hBD-2 and hBD-3 expression. On the other hand the induction of a TH1 response, i.e. TNF-α and interferon-γ might directly induce or enhance the expression of hBDs as reported recently [47]. On the other hand PMNs are able to establish an anti-fungal response by upregulate the expression of TLR4 in epithelial cells [5]. The observed more pronounced effect on hBD-3 expression are in line with the theory that hBD-3 might be clinically more relevant than hBD-2 since hBD-2 and hBD-3 have potent fungicidal activity against *C. albicans *at micromolar concentrations, with hBD-3 being about 10 times more fungicidal than hBD-2 [16,17,19]. Finally we demonstrated that hBD-3 expression was induced by transactivation of the EGFR independent of EGF (Figure 8 and 9). Many signals besides EGF converge and result in EGFR-dependent signaling which is important for various biological processes including normal growth, development and as shown recently for inflammation or innate immune response [48,49]. Using blocking antibodies for the EGFR-ligands EGF, HB-EGF, amphiregulin and TGF-α we were able to show that most likely TGF-α is involved in the EGFR mediated upregulation of hBD-3 expression (Figure 9). Since we are also able to inhibit the induction of hBD3 by the supernatants of 1 × 10^5 ^*C. albicans *cells/ml by EGFR and/or TGF-α blocking antibodies we speculate that the EGFR-ligand is secreted by OE21 cells. Ligands of the EGFR are expressed as transmembrane precursors. These are released from the cell surface following shedding of the extracellular domain by a family of metalloproteinases (a disintegrin and metalloprotease (ADAM)). ADAM10, -12 and -17 are the sheddases of the EGFR ligands in response to various stimuli. Since human cathelicidin cationic antimicrobial protein (hCAP)-18 and its active peptide LL-37 have been shown to be involved in the transactivation of the EGFR at the airway epithelial surfaces [50,51] it is tempting to speculate that the activation of PMNs by *C. albicans *leads to secretion of proinflammatory mediators including leukotriene B4 (LTB4) and LL-37 [52,53] which in turn amplifies the inflammatory response [54,55] and leads to the shedding of EGFR-ligands.

In conclusion we were able to establish a complex *in vitro *model for *Candida *infection of esophageal cells to investigate the signalling events leading to upregulation of hBD-2 and hBD-3 expression.

## Conclusion

This analysis of signal transduction events in esophageal *Candida *infection demonstrated a potential functional interaction of epithelial cells with PMNs and that EGFR, NF-κB and MAPK/AP-1 are involved in divergent signalling events governing hBD-2 and hBD-3 expression. Especially the observed effect of PMNs on hBD expression might explain the high incidence of *Candida *esophagitis and *Candida *related deaths in neutropenic patients.

## Methods

### Materials

Cell culture medium was purchased from PAA-Labs (Linz, Austria), fetal bovine serum from Seromed (Berlin, Germany), recombinant TNF-α from Sigma. Supershift and Westernblot p65, p50 and c-Jun antibodies were from Santa Cruz Biotechnology (Santa Cruz, CA).

### Organism and Cell culture

OE21 cells, a moderately differentiated oesophageal squamous carcinoma cell line were cultured in RPMI 1670 supplemented with 10% fetal calf serum and divided every 3 days. Cells for experimental purposes were cultured in 6 well cell culture plates with the same medium and used at subconfluence. C. albicans (a clinical isolate from oesophagitis [25]) was taken fom a frozen strain suspension (BHI, glycerine). 10 μl was transferred into 10 ml TSB-Bouillon and incubated overnight at 37°C. The cells were then separated from the medium by centrifugation at 2800 × g for 5 min, washed two times with PBS, and finally resuspended in RPMI for each experiment. The final concentration of C. albicans was 1 × 10^4^/ml.

### Inhibitor studies

OE21 cells were pre-treated with specific inhibitors or neutralizing antibodies for 30–60 min prior to and during *Candida *or cytokine stimulation. The neutralzing antibodies against the EGFR (Upstate, USA), TGF-α (R&D Systems), HB-EGF (R&D Systems), amphiregulin (R&D Systems), and EGF (Pepro Tech, Germany) were used at the indicated concentrations. Inhibitors utilized were: PD98059 (Calbiochem) at 10 μM, SB203580 (Tocris, Ellusville, USA) at 10 μM, SP600125 (Tocris, Ellusville, USA) at 20 μM. Inhibitor were disolved in DMSO and DMSO was used as a vehicel control.

### Generation of Supernatants from *C. albicans*

To analyze the effects of secreted factors from *C. albicans *on OE21 cells, *C. albicans *were cultured with respective medium for 48 h in the indicated density (0.5 × 10^4 ^to 1 × 10^5 ^cells/ml). Before use, these conditioned media were precleared by centrifugation (10,000 rpm for 10 min) and used for additional experiments.

### Isolation of polymorphonuclear leukocytes (PMN)

PMN were purified using LeucoSep tubes according to the instructions of the manufacturer (Greiner Bio-One). In brief, 3 ml of Ficoll-Paque was preloaded in a 14 ml LeucoSep tube by centrifugation for 30 s at 1,000 g. The heparinized whole-blood samples of healthy volunters were diluted with equal volumes of PBS, and 6 ml of the diluted blood was added to a LeucoSep tube. The cell separation tubes were centrifuged for 15 min at 800 g without braking at room temperature. The cell suspension was collected, and the cells were washed twice in PBS (for 10 min at 640 and 470 g, respectively, for the two successive wash steps) and resuspended in complete RPMI medium before counting.

### Stimulation with Supernatants, direct cell contact and transwell coculture model

2 × 10^5 ^OE21 cells were seeded into a six-well culture plate. For stimulation experiments medium was replaced after 24 h with the *C. albicans *supernatants and cultured for indicated times. For direct cell contact suspension cultures of *C. albicans *(final concentration 0.5 × 10^4 ^cells/ml) or PMNs (final concentration 0.5 × 10^6 ^cells/ml) were added to the OE21 cells after initial 24 h culture and incubated for the indicated times. In the transwell setting (for a schematic representation please refer to Figure 2) 2 × 10^5 ^OE21 cells were cultured into the bottom compartment of a six-well culture plate. After 24 h PMNs or *C. albicans *were added to the bottom compartment and the corresponding cell type was seeded into the top transwell compartment (Costar GmbH, Bodenheim, Germany) and cultured for additional 24 h.

### RNA isolation and cDNA synthesis

Total RNA was isolated using the RNeasy Kit from Qiagen (Hilden, Germany) and reverse-transcribed into single-stranded cDNA, as described previously [56].

### Primer

The oligonucleotide sequence and product size for each primer pair used were as described previously [25].

### Quantification of gene expression by real time PCR

Real-time PCR analyses were performed as previously described [56]. Standard curves for each mRNA were constructed by cloning the purified PCR-products containing the target sequence into pCR-Blunt II-TOPO vector (Invitrogen). Concentration of the reference plasmid was measured spectrophotometrically and transformed into number of copies/μl by calculation. The absolute mRNA transcript number in each sample was calculated by use of calibration curves. Identical results were obtained in control experiments.

### Cytokine profilling

Culture supernatants were cleared by centrifugation (6.000 rpm, 5 min, 4°C) and protein concentration was determined by the BioRad assays. For a qualitative screening for the cytokine content, samples were adjusted to equal protein concentration and submitted to cytokine antibody arrays (Cytokine Profiler kit, R&D systems) following the manufacturer's instructions.

### siRNA transfection

For knock down of RelA/p65, cells were seeded into 6 well plates (2 × 10^5 ^cells/well) and grown overnight, then transfection with 12 μl/well RNAiFect reagent (Invitrogen) and 2 μg/well of either Stealth negative control siRNA (Invitrogen) or Stealth RelA/p65 siRNA (Invitrogen) was performed for 48 h.

### Western Blotting

Cellular lysates were prepared and western blotting was performend as described previously [57,58].

### EMSA

Nuclear extracts were prepared as described previously [58] and incubated with a γ32P-labelled oligonucleotide containing a consensus NF-κB-binding or consensus AP-1 site (Promega; Mannheim, FRG). After 30 min incubation at room temperature, samples were separated by gel electrophoresis at 100 V and 4°C. Gels were dried and exposed to X-ray Hyperfilm (Amersham; Freiburg, FRG).

### Statistics

Data are presented as mean ± SD and analyzed by Student's t-test. A p-value < 0.05 (indicated as * in the figures) was considered as statistically significant.

## Authors' contributions

NS, GB, RP and KK carried out the experimental studies and drafted the manuscript. KR, RP, KHH, SS, URF and PR participated in the design of the study and performed the statistical analysis. AA conceived of the study, and participated in its design and coordination and helped to draft the manuscript. All authors read and approved the final manuscript.

## Supplementary Material

Additional file 1 **PMNs induce IL-6 and IL-8 in OE21 cells**. Supernatants of OE21 cells kept in culture alone (upper panel) or with with direct contact with 0.5 × 10^6 ^PMNs/ml (lower panel) for 24 h were submitted to a peptide arrays detecting a broad panel of chemo- and cytokines. Click here for file
