# Supplementary figures and images for: The expression of the β-defensins hBD-2 and hBD-3 is differentially regulated by NF-κB and MAPK/AP-1 pathways in an in vitro model of Candida esophagitis
Source: BMC Immunol. 2009 Jun 12;10:36. doi: 10.1186/1471-2172-10-36 (PMC2702365; doi:10.1186/1471-2172-10-36)

Cytokine expression OE21 cells alone

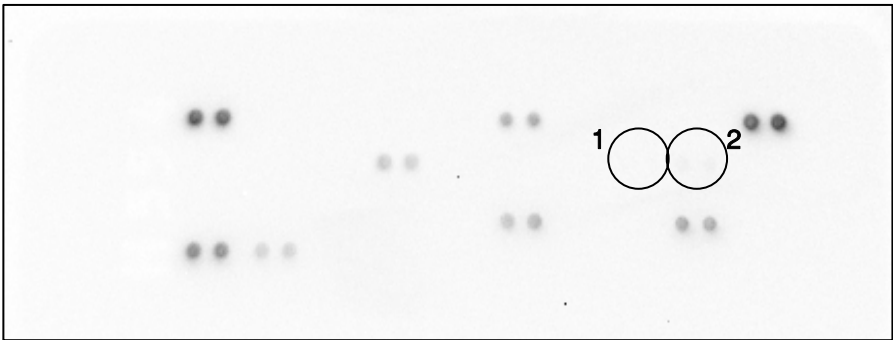

1 IL-6  
2 IL-8

Cytokine expression OE21 cells + PMN

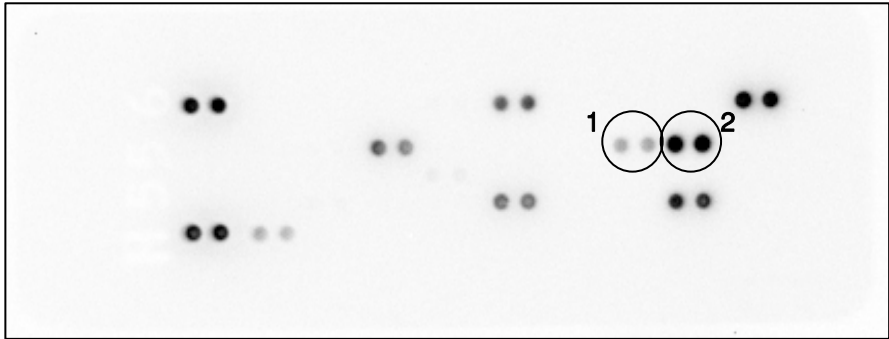

1 IL-6  
2 IL-8

Supplement: Additional file 1 — PMNs induce IL-6 and IL-8 in OE21 cells. Supernatants of OE21 cells kept in culture alone (upper panel) or with with direct contact with 0.5 × 10^6^ PMNs/ml (lower panel) for 24 h were submitted to a peptide arrays detecting a broad panel of chemo- and cytokines. [file 1471-2172-10-36-S1.pdf]
